# Cardiopulmonary Exercise Testing and Cardiac Biomarker Measurements in Young Football Players: A Pilot Study

**DOI:** 10.3390/jcm11102772

**Published:** 2022-05-14

**Authors:** Alexandru-Dan Costache, Mihai Roca, Cezar Honceriu, Irina-Iuliana Costache, Maria-Magdalena Leon-Constantin, Ovidiu Mitu, Radu-Ștefan Miftode, Alexandra Maștaleru, Dan Iliescu-Halițchi, Codruța-Olimpiada Halițchi-Iliescu, Adriana Ion, Ștefania-Teodora Duca, Delia-Melania Popa, Beatrice Abălasei, Veronica Mocanu, Florin Mitu

**Affiliations:** 1 Department of Internal Medicine I, Faculty of Medicine, University of Medicine and Pharmacy “Grigore T. Popa”, 700115 Iasi, Romania; adcostache@yahoo.com (A.-D.C.); irina.costache@umfiasi.ro (I.-I.C.); leon_mariamagdalena@yahoo.com (M.-M.L.-C.); mituovidiu@yahoo.co.uk (O.M.); radu.miftode@yahoo.com (R.-Ș.M.); alexandra.mastaleru@gmail.com (A.M.); iliescud@gmail.com (D.I.-H.); adriana.ion@hotmail.com (A.I.); stefaniateodoraduca@gmail.com (Ș.-T.D.); deliamelaniapopa@gmail.com (D.-M.P.); mitu.florin@yahoo.com (F.M.); 2 Department of Cardiovascular Rehabilitation, Clinical Rehabilitation Hospital, 700661 Iasi, Romania; 3 Faculty of Physical Education and Sports, “Alexandru Ioan Cuza” University, 700115 Iasi, Romania; chonceri@yahoo.fr (C.H.); beatrice.abalasei@uaic.ro (B.A.); 4 Department of Cardiology, “St. Spiridon” Emergency County Hospital, 700111 Iasi, Romania; 5 Department of Cardiology, Arcadia Hospital, 700620 Iasi, Romania; 6 Department of Mother and Child Medicine-Pediatrics, University of Medicine and Pharmacy “Grigore T. Popa”, 700115 Iasi, Romania; codrutzache@yahoo.co.uk; 7 Department of Pediatrics, Arcadia Hospital, 700620 Iasi, Romania; 8 Department of Morphofunctional Sciences II, Faculty of Medicine, University of Medicine and Pharmacy “Grigore T. Popa”, 700115 Iasi, Romania; veronica.mocanu@gmail.com

**Keywords:** athletes, cardiopulmonary exercise testing, cardiac biomarkers

## Abstract

Constant and intense physical activity causes physiological adaptive changes in the human body, but it can also become a trigger for adverse events, such as sudden cardiac arrest or sudden cardiac death. Our main objective was to assess the use of combined cardiopulmonary exercise testing (CPET) and cardiac biomarker determinants in young professional athletes. We conducted a study which involved the full examination of 19 football players, all male, aged between 18 and 20 years old. They underwent standard clinical and paraclinical evaluation, a 12-lead electrocardiogram (ECG), and transthoracic echocardiography (TTE). Afterwards, a tailored CPET was performed and peripheral venous blood samples were taken before and 3 h after the test in order to determine five biomarker levels at rest and post-effort. The measured biomarkers were cardiac troponin I (cTnI), myoglobin (Myo), the MB isoenzyme of creatine-kinase (CK-MB), the N-terminal prohormone of brain natriuretic peptide (NT-proBNP) and D-dimers. While cTnI and NT-proBNP levels were undetectable both at rest and post-effort in all subjects, the variations in Myo, CK-MB and D-dimers showed significant correlations with CPET parameters. This highlights the potential use of combined CPET and biomarker determinants to evaluate professional athletes, and encourages further research on larger study groups.

## 1. Introduction

Regular physical activity has many beneficial effects with regard to the cardiovascular system: it induces a physiological remodeling of the heart and results in molecular and cellular adaptive changes, which themselves have a cardioprotective effect. The long-term and moderate practice of sports, especially aerobic–isotonic ones, are even encouraged in those with chronic cardiac diseases [1]. Even in the older population, regular physical activity has been shown to slow degenerative cardiac changes and significantly improve quality of life [2].

Concerning young athletes, there are both morphological and functional adaptations of the heart in relation to sustained and intense physical activity. Morphologically, the myocardium suffers a process of hypertrophy and the chambers become dilated, as to adapt to the increased hemodynamic stress. The main difference between these physiological adaptative changes and the pathological ones, which are encountered in chronic cardiac diseases, lies in the fact that hemodynamic stress is intermittent in physical activity, whereas in chronic cardiac disease it is continuous. These structural modifications, especially the enlargement of the chambers, together with the increased vagal tonus, are the ones that, in turn, lead to electrophysiological modifications [3].

Most of the electrophysiological changes, in the context of constant physical activity, which have an electrocardiographic (ECG) expression, can be interpreted as within the normal limits without warranting further investigation. However, they are not to be confused with pathological ECG patterns, which have an underlying cardiac pathology and which can act as triggers for adverse events, such as sudden cardiac death [4,5] (see Table 1).

The 2020 European Society of Cardiology (ESC) guidelines on sports cardiology and exercise in patients with cardiovascular disease are a milestone in both the field of cardiology and sports medicine. As the first edition, they introduce numerous up-to-date information and recommendations for the practice of physical exercise in both healthy individuals and persons with known cardiovascular pathologies. Among the potential adverse cardiovascular events, the two most dangerous are sudden cardiac arrest (SCA) and sudden cardiac death (SCD). As tragic as they may be, especially if occurring in young healthy individuals, SCA and SCD are not reported in all countries; therefore, their incidences cannot be accurately estimated. However, according to most recent statistics, SCA is diagnosed in 1 out of 80,000 high-school-aged athletes and in 1 out of 50,000 college-aged athletes. Based on gender, ethnicity, and type of sports practiced, the most at-risk group is represented by male, black, basketball athletes in the United States, or football athletes in Europe [6,7,8,9,10,11] (see Table 2).

The most important etiology of SCD in young athletes is congenital cardiac structural disorders; yet, a significant number of autopsies (44%) cannot identify a cause, these being the autopsy-negative sudden unexplained deaths (AN-SUD) [6,7,12,13,14,15].

The current guidelines recommend, for the routine screening for cardiovascular disease in young athletes, the patient’s history, a physical examination, and a 12-lead standard ECG, which actually outweighs the first two. Although the cardiac ultrasound could offer significantly more information, especially regarding the congenital anomalies, a lack of evidence restricts its implementation in the standard screening protocols [6,16,17,18,19,20,21,22].

There are a few methods that allow the assessment of cardiorespiratory fitness (CRF) in youths which are currently used, such as the 20-m shuttle run test (20mSRT). It is a feasible, reliable, and easy to put in practice method of evaluation, and is currently being discussed to be included in the standardized evaluation protocols [23].

Given the wide spectrum of parameters evaluated (pulmonary, cardiovascular, muscular and oxidative) and the multitude of data and correlations it provides, the cardiopulmonary exercise testing (CPET) is an essential tool in current practice [24]. It is also useful in healthy individuals and in the athletic performance assessment, as maximal oxygen uptake (VO_2_ max) is directly linked to the exercise capacity [25,26]. Of course, normal parameters vary, as elite aerobic athletes can reach VO_2_ max values over 80 mL/kg/min, compared to untrained individuals who are in the 30–45 mL/kg/min range [27]. The test can be performed on a treadmill (mostly in the United States) or on the cycle ergometer (mostly in Europe). The cycle ergometer testing has a low associated cardiovascular risk in evaluating young healthy athletes [28,29] (see Table 3).

A particular role of CPET in evaluating athletes is to differentiate between their physiological left-ventricular hypertrophy (LVH) and hypertrophic cardiomyopathy (HCM) [30,31].

**Table 3:** CPET indications (adapted from Löllgen et al. [31]).

CPET Indications			References
Asymptomatic individuals	Patients	Follow-up assessment during training	[31]
Latent disease diagnosis Risk factor identifications Physical performance ability assessment Guidance/monitorization of training	Cardiac/respiratory disease diagnosis Symptom evaluation	Training regimen recommendations	

Cardiac troponins (cTn) are regulatory components of the cardiac muscle contraction. Cardiac troponin I (cTnI) is of interest due to its phosphorylation sites, which regulate different contractile responses [32,33]. It has been shown that physiological left ventricular hypertrophy, which is specific to trained persons, causes a reversible increase in Ca^2+^-dependent force production and in Ca^2+^-sensitivity in left ventricular (LV) cardiomyocytes. Together with a reduction in cTnI phosphorylation, it is suggestive for an adaptive measure and for the preserved or even increased contractile function, despite the morphology changes [34]. In contrast, untrained persons who are subjected to a sudden physical effort do register increases in cTnI levels, although they are not pathological [35]. Additionally, of interest is the fact that peak levels of hs-cTn are registered between 3 and 4 h after peak physical stress [36].

Myoglobin is a biomarker produced by both the myocardial and skeletal muscle cells. Therefore, its levels rise in cardiac diseases (myocarditis, acute coronary syndromes) as well as muscular stress or strenuous physical effort. It can aid the differential diagnosis of cardiac or muscular damage, whether its levels rise independently or in conjunction with other cardiac biomarkers. Its use in establishing the diagnosis of acute coronary syndrome is low in the absence of other biomarkers’ elevation (CK-MB, cTn); however, it is the one whose levels rise earliest in the serum [37,38,39].

The MB isoenzyme of creatine-kinase (CK-MB) is specific to the cardiac muscle. On its own it cannot predict the cardiovascular risk accurately, unless it is associated with the rise of cTn; thus, its use is still being debated. Its practical utility resides in situations where the estimated glomerular filtration rate (eGFR) is below 15 mL/min/m^2^, or in recent-onset acute coronary syndromes [40,41].

The N-terminal prohormone of brain natriuretic peptide (NT-proBNP) levels do increase during intense physical exercise, especially in non-trained, amateur athletes. Yet, when not correlated with ECG changes, they are not significant for a cardiovascular pathology [35]. Its release in the blood is more associated with aging and with the effort-generated hypertensive stress on the ventricular and atrial walls [42,43,44]. Of course, it is essential in diagnosing potential ischemic changes in the myocardium, especially in conjunction with cTn, and with proven benefit in risk stratification in athletes [45,46,47].

While not being specific markers of myocardial cytolysis, D-dimers levels reflect intravascular fibrinolysis and show higher concentrations in patients with severe atherosclerosis or other causes of coronary stenosis [48]. They have more use in confirming or infirming venous thromboembolism, acute aortic dissection, cardioembolic stroke, or left atrium thrombosis in patients with atrial fibrillation [49]. Additionally, an independent rise in their levels can be specific in persons with implantable cardiac devices [50].

The aim of our study was to assess the relationship between effort-induced cardiac biomarker variations and CPET parameters, and their usefulness in the evaluation of young athletes.

## 2. Materials and Methods

### 2.1. Experimental Approach

We conducted a complete cardiovascular evaluation of professional football players, with an emphasis on cardiopulmonary exercise testing (CPET) and blood biomarkers (cTnI, myoglobin, CK-MB, NT-proBNP and D-dimers) in order to evaluate and assess the cardiovascular risk. The study was designed following the recommendations for clinical research contained in the Helsinki Declaration of the World Medical Association, and the protocol was approved by both the Ethics Committee of the Clinical Rehabilitation Hospital in Iași, Romania, approved on 24 March 2021, and the Ethics Research Committee of the “Grigore T. Popa” University of Medicine and Pharmacy in Iași, Romania, nr. 72, approved on 25 April 2021.

### 2.2. Participants and Protocol

We evaluated professional football players (n = 19). Football club trainers from the region of Moldova in the counties of Iași and Vaslui were informed of the ongoing study and were given our contact data. They themselves informed the registered football players aged between 18 and 20 years, and the first 30 to voluntarily contact us were automatically selected to be evaluated. In order for them to be included, they had to participate actively in regular training during the season of the last year, or at least the last months. Those who had missed part of the last months of training or playing due to injury, or who were in the recovery period after an injury of any sort, were excluded, as well as those with cardio-pulmonary pathological findings during the initial evaluation.

The participants were all male, aged between 18 and 20 years old (mean 18.47 ± 0.841), and fully informed about the procedures, research, and protocols used. They were asked to halt any strenuous training sessions or games a minimum of 24 h before the evaluation, and not to consume any foods or beverages other than water before the initial blood sampling, so as not to interfere with the blood sugar values. Afterwards, they could have a small snack or light meal at least an hour before the CPET. They were admitted through day hospitalization in August and September 2021. Upon admission, they filled out the informed consent forms regarding all the procedures and their inclusion in the study, and underwent rapid COVID-19 antigen testing, which, if negative, would allow them to further proceed with the investigations. They underwent a clinical and paraclinical evaluation with an emphasis on the cardiovascular system, followed by a 12-lead resting ECG, TTE, and CPET. Blood samples for the measurements of cardiac biomarkers values were taken at rest before the procedures and 3 h after finishing the CPET.

### 2.3. Initial Evaluation

After the initial blood samples were taken, a complete clinical evaluation was conducted with an emphasis on the cardiovascular system with its four major components (inspection, palpation, percussion and auscultation). The resting blood pressure (BP) and heart rate (HR) values were measured comparatively on the both upper limbs and in the orthostatism using a Rossmax X3 BT automatic blood pressure monitor with a brachial cuff. Height and weight were measured on a SECA digital measuring station.

### 2.4. Resting ECG

A standard 12-lead resting ECG was performed on a BTL-08 LC device, both in post-expiratory apnea and in deep post-inspiratory apnea, and every morphological change was analyzed to be considered as either normal for an athlete or pathological (see Table 1), and possibly contraindicate further procedures.

### 2.5. Cardiac Ultrasound

The standard transthoracic echocardiographic evaluation was performed on a Toshiba Aplio device, prior to the CPET, to assess the cardiac function and to exclude any possible contraindications, using the M-mode, pulse wave Doppler (PWD), continuous wave Doppler (CWD), and color Doppler methods, according to the European Society of Cardiology (ESC) and European Association of Cardiovascular Imaging (EACVI) protocols.

### 2.6. Cardiopulmonary Exercise Testing

Functional capacity was assessed by cardiopulmonary exercise testing (CPET) on the BTL CardioPoint software (version 2.32 manufactured by BTL Industries Ltd., Herfordshire, UK) and the BTL-compatible device. We used a progressive maximal symptom-limited CPET protocol on the cycle ergometer, specifically tailored for the athletes: they started on a workload of 15 Watts which was set to increase every 30 s with 12.5 Watts. The duration of the testing was between 10 and 12 min and the recovery period was 10 min.

The most important CPET parameters were: maximal work rate (absolute value, WR (Watt) and percentage of the predicted value, WR% (%)); oxygen uptake with maximal aerobic capacity (absolute value, VO_2_ max (mL per min) and percentage of the predicted value, VO_2_ max%); carbon dioxide output (VCO_2_ (mL per min)); oxygen uptake at the anaerobic threshold (AT) (mL per min); peak value of the respiratory exchange ratio (RER) defined as the ratio between VCO_2_ and VO_2_; maximal heart rate (HR (bpm)); O_2_ pulse as the ratio of VO_2_ to heart rate, reflecting the amount of O_2_ extracted per heartbeat; ventilatory efficiency expressing the rise in minute ventilation (VE) relative to VCO_2_ (VE/VCO_2_ slope); and heart rate reserve (difference between maximal HR and resting HR, HRR (bpm)). VO_2_, VCO_2_, and AT were also expressed as values normalized by body weight (mL per min per km).

Metabolic efficiency was assessed by measuring the increase in VO_2_ over the rate of increase in work rate (ΔVO_2_/ΔWR). The slope of this relationship expresses the ability of the muscle to extract O_2_ during exercise.

Blood pressure was monitored every 2 min using the auscultatory method, while a real-time 12-lead ECG was recorded.

To clinically determine the intensity of the exercise, a subjective rating of the intensity of perceived fatigue was determined by a 6 to 20 Borg scale of perceived exertion.

The test was halted, according to current recommendations, when the subject requested it, upon symptoms or fatigue occurrence, when the blood pressure (BP) measurement exceeded 220 mmHg for the systolic value, or 120 mmHg for the diastolic value, or when suggestive ischemic ECG patterns appeared.

### 2.7. Cardiac Biomarker Determination

Peripheral venous blood was collected at rest for the basic laboratory parameters and also to determine the mentioned biomarker values at rest. Three hours after finalizing the CPET, another sample was taken to measure the variations of the biomarker values after the stress test, so as to allow them time to appear and reach certain levels in the blood. All blood sampling was taken from the antecubital veins and in kept dedicated vacutainers (with sodium citrate for D-dimers and lithium heparin for CK-MB, Myo, cTnI, and NT-proBNP). Their measurements were performed immediately after, using whole blood on the FIA 8000 device from Getein Biotechnology Co. Ltd. on its dedicated panels: triple tests for CK-MB, myoglobin, and cTnI (CK-MB: measuring range 2.5–80 ng/mL, lower detection limit ≤ 2.5 ng/mL, recovery 96%; myoglobin: measuring range 30–1000 ng/mL, lower detection limit ≤ 30 ng/mL, recovery 95%; cTnI: measuring range 0.5–50 ng/mL, lower detection limit ≤ 0.5 ng/mL, recovery rate 95%, measuring time 15 min), double tests for cTnI and NT-proBNP (cTnI: measuring range 0.5-50 ng/mL, lower detection limit ≤ 0.5 ng/mL, recovery rate 95%; NT-proBNP: measuring range 10–12,000 pg/mL, lower detection limit ≤ 100 ng/mL, recovery 99%, measuring time 18 min) and a single test for D-dimers (D-dimers: measuring range 0.1–10 mg/L, lower detection limit ≤ 0.1 mg/L, recovery rate 99%, measuring time 7 min) were completed. All the assays were stored, according to manufacturer specifications, at a temperature between 4 and 30 degrees Celsius and used before the expiration date was reached and within 1 h since each foil was opened.

### 2.8. Data analysis and Statistics

Data analysis was performed using SPSS 20.0 (Statistical Package for the Social Sciences, Chicago, IL, USA). Data were presented as mean ± standard deviation (SD), or as median with an interquartile range for continuous variables. These variables were compared by the non-parametric Mann–Whitney U test, considering that their distribution did not satisfy the assumption of normality, due to the small number of subjects included in the study. For the same reason, correlations between continuous variables were assessed by calculating the Spearman correlation coefficients. A two-sided *p*-value < 0.05 was considered significant for all analyses.

## 3. Results

### 3.1. Anthropometric Data and Initial Clinical and Paraclinical Evaluation

During the clinical evaluation, no significant pathological findings were encountered. The resting systolic blood pressure (SBP), diastolic blood pressure (DBP), and heart rate (HR) were within the normal range and no significant BP differences were noticed between the upper limbs or in the orthostatic position. Using the measured height and weight values, the body mass index (BMI) was calculated using the formula weight/(height)^2^. Most of them had a normal BMI (18.55–24.99 kg/m^2^), while four of them had a BMI of 25 kg/m^2^ or above. Therefore, we measured the abdominal circumference and none of them had a value above 102 cm (see Table 4.).

### 3.2. 12-Lead Resting ECG

All subjects were in sinus rhythm and 14 had sinus bradycardia (HR < 60 bpm), while the other 5 had normal baseline HR. Two athletes had a right-heart axis deviation, and one had a 90-degree axis. Four of them had normal morphology, while in fourteen a high QRS-complex voltage was encountered, three showed a complete RBBB (RSR’ pattern in the V1 and V2 leads and a QRS duration >0.12 s), and three displayed an incomplete RBBB (RSR’ pattern only in the V1 lead and a QRS duration <0.12 s). By analyzing these patterns, the increased QRS complex voltage, the incomplete RBBB and the sinus bradycardia were considered to be normal for an athlete, while the complete RBBB and the right-axis deviation were considered borderline changes (see Table 1). However, they were not considered to be a reason to contraindicate further investigations.

### 3.3. Transthoracic Cardiac Ultrasound

The TTE parameters were within the normal limits and no significant pathological encounters were made: two subjects had a mild tricuspid valve regurgitation, two had an anterior mitral valve prolapse, and two had a mild mitral valve regurgitation. However, these findings were considered benign and not a reason to contraindicate further investigations (see Table 5).

### 3.4. CPET

In 12 out of 19 subjects, the CPET had to be halted due to the recorded systolic BP values of 220 mmHg or higher, while in the remaining 7, the test was stopped due to muscle fatigue. No ECG ischemic patterns were identified, nor any symptoms such as angina were declared. Eleven of them did not reach the 85% heart rate threshold, yet only two had a peak VO_2_ value below 85% of the predicted. The respiratory exchange ratio (RER) was, in all cases, over 1.00 and only in three cases below 1.0 (see Table 6).

### 3.5. Cardiac Biomarkers

cTnI and NT-proBNP levels were below the lower detection limit of the assays in the blood samples in all participants, both at rest and 3 h after the CPET, while seven of the athletes had values below the lower detection limit of the assays for all biomarkers. In four subjects, myoglobin levels increased after 3 h compared to the at-rest values, while in three subjects they decreased. In two participants, CK-MB levels increased post-CPET, while in one athlete they decreased. The D-dimer levels decreased after CPET in five subjects, while in three the values increased. For the statistical analysis, all participants were considered and for those with undetectable values, the lower detection limit value for each biomarker was assigned (see Table 7).

### 3.6. Interrelations between Cardiac Biomarkers and Functional Parameters

**Figure 1:**
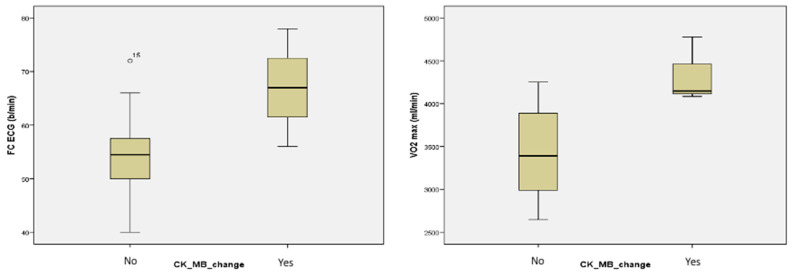
Significant differences between the groups without changes (No) and with changes (Yes) in CK-MB values on resting ECG heart rate (**left**) and on CPET VO_2_ max (**right**), respectively.

Statistically, in the Mann–Whitney nonparametric tests, CK-MB changes showed significant differences in the heart rate (HR) on the resting ECG (*p* = 0.047), the VO_2_ max at the CPET (*p* = 0.008), and with the peak load (WR max) on the CPET (*p* = 0.014) between those with and those without changes in CK-MB levels (see Table 8 and Figure 1).

**Figure 2:**
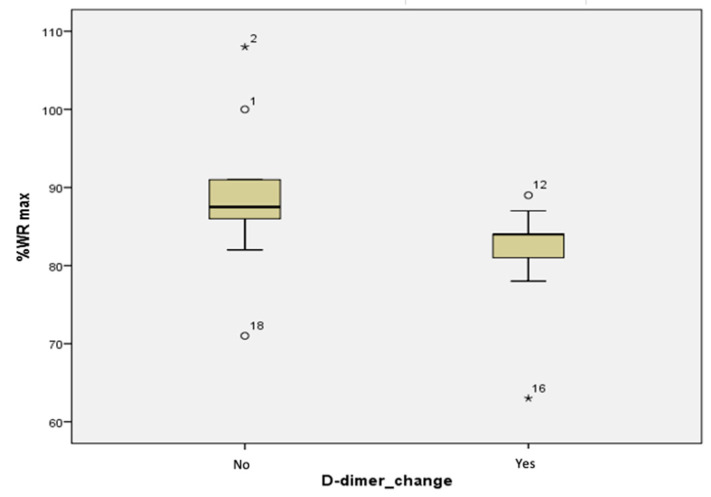
Significant differences between the groups without changes (No) and with changes (Yes) in D-dimers values on CPET %WR max.

D-dimers showed significant differences within the %WR max on the CPET (*p* = 0.035), between those with and those without changes in D-dimers levels (see Table 9 and Figure 2).

**Figure 3:**
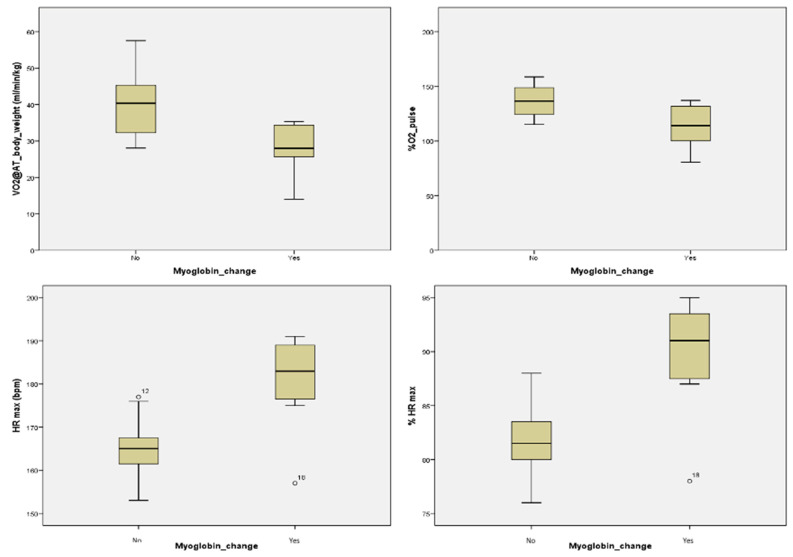
Significant differences between the groups without changes (No) and with changes (Yes) in myoglobin values.

Myoglobin showed significant differences between those with and those without changes in myoglobin levels for E/A and A/E ultrasound parameters (*p* = 0.036), VO_2_ at the anaerobic threshold (VO_2_ @ AT) on CPET (*p* = 0.045), and with VO_2_ @ AT/body weight on CPET (*p* = 0.017). Additionally, significant differences were observed with % Watt max on CPET (*p* = 0.045), with % O_2_ pulse on CPET (p=0.028) and with HR max (*p* = 0.013) and % HR max (*p* = 0.017) on CPET (see Table 10 and Figure 3).

## 4. Discussion

Laboratory explorations are gaining more and more ground and sports cardiology represents the new trend in medicine. Therefore, as confirmed by previous studies, their combination is an opportunity for future research [51].

The initial evaluation showed findings consistent with athletes with a normal BMI. In the few cases where BMI was over 25 kg/m^2^, this was due to the higher muscle mass, as the abdominal circumference was normal. ECG interpretation also showed heart rate and morphology patterns which are considered normal in an athletic population as part of the effort adaptation process (sinus bradycardia, right bundle branch block). The cardiac ultrasound highlighted parameters of increased cardiovascular performance, such as a higher ejection fraction and a mild left ventricular hypertrophy, which, once again, is part of the adaptation to effort process in athletes.

In addition, cardiorespiratory functional evaluations are becoming more used in current practice and screening. As obesity and the sedentary lifestyle are becoming more prevalent, especially among the young population, cardiorespiratory functional evaluations are useful tools for assessing the fitness level. In recent studies, CRF has been proven to correlate negatively with parameters such as BMI and the sedentary lifestyle at younger ages [52].

A study published by Olekšák et al. involved the evaluation of CPET on young Slovenian footballers. They compared the CPET parameters on children and adolescents and concluded that some of them were physiologically higher in athletes and with growing age (VO_2_ max, Watt max) [53].

The CPET parameters evaluated on the 19 subjects were comparable with those in other studied athlete cohorts. It is interesting to note that some of the registered values, such as a lower VO_2_ max than predicted, were consistent with the tests which were halted for high BP values. This once again proves the utility of this particular evaluation and shows how physical performance CRF can be limited by an abrupt rise in BP.

Other investigations whose utility has been confirmed in recent studies include biomarker measurements. In a paper published by Mahanty et al., cTn, BNP and hypoxanthine were proven as means of assessing the cardiovascular impact of intense physical activity. Therefore, they are being considered to be implemented in the future as part of screening protocols [54]. 

A metanalysis published in 2015 concluded that cTnT, hs-cTnT, BNP, NT-proBNP, and D-dimers do suffer serum level changes when a person is performing a high-intensity physical effort, which may interfere with their interpretation in an emergency unit when an acute coronary syndrome, heart failure or pulmonary embolism can be suspected [55]. 

When investigating young football players, after a full-time football match, cTnI and NT-proBNP levels rose above the baseline and remained elevated even 24 h after the game, yet they never reached pathologically significant values. This rise, compared to our study can be attributed to the higher intensity and duration of the football match (90 min, on average) compared to a CPET [56]. Similarly, blood samples taken from participants in the 2016 Barcelona marathon showed an increase in NT-proBNP levels; however, the intensity of the physical stress and its duration were considerably higher than during a CPET [44].

Another study published in 2021 included individuals that participated in 2018 in the North Sea Race, a 91 km leisure sport mountain bike race. Prior to the race, they performed a CPET. Their cTnI levels were measured before, 3 h, and 24 h after both the race and CPET. The peak values were reached at the 3-h mark, though it should be once again mentioned that, in both cases, the duration and intensity of the physical exercise were higher than in the CPET which we conducted. However, a noticeable inter-individual variation was also observed [57].

There are several mechanisms incriminated for this pattern of variation in biomarker blood levels, such as microvascular ischemia, deficiencies in cardiac metabolism, a systemic inflammatory surge, or even an impaired renal function during intense exercise [58]. This particular dynamic of cTnI concentrations, with an early peak followed by a rapid normalization within hours (maximum 48–72 h), renders an active myocyte necrosis highly improbable, and rather suggests the above-mentioned secondary mechanisms. In our study, including highly trained athletes, we did not observe any variation of cTnI or NT-proBNP, compared to baseline. These somehow atypical kinetics can be explained by the rather short duration of the CPET, performed by apparently healthy, well-trained individuals, without the deleterious effects of prolonged and exhausting sports that can presumably represent important triggers for the release of cardiac biomarkers. This finding is consistent with the results of Marshall et al., who recently highlighted a similar pattern of troponin fluctuation, with significant variations compared to baseline occurring only in subjects who performed a moderate or intense training regimen. Very interestingly, the same authors noted that a shorter duration of high-intensity exercise induced a more important increase in troponin compared to prolonged, but less intensive, training [59]. Basically, these heterogeneous patterns outline the importance of the duration, intensity, and type of training when assessing cardiac biomarkers. A promising future scenario also assumes the use of novel cardiac biomarkers, such as the soluble suppression of tumorigenesis-2 (sST2) for the early detection of subclinical myocardial injury during sports. Being a marker of increased myocardial strain, fibrosis, and neurohormonal activation, sST2 exhibits a superior prognosis value compared to NT-proBNP or cTnI in patients presenting acute myocardial injury [60,61].

Recent studies have shown that cardiac biomarkers have an important negative predictive value. Thus, low or undetectable hs-cTnI levels can help exclude an inducible myocardial ischemia, in both patients with known coronary artery disease (CAD) and patients without [62,63,64].

Comparing male and female football players who had CK-MB values measured before, immediately after, and 15 min after a running training session, researchers described a slight increase in CK-MB values immediately post-exercise, most notably in the women’s group, yet these values returned to baseline at the 15 min timepoint. The groups comprised both genders and the measurements were performed at different timepoints (immediately after and 15 min after). This study was comparable to ours with regard to the type of sports practiced and the number of participants [65].

Another study measured myoglobin and CK levels immediately after, at the 24 h, 48 h and 72 h timepoints after a high-intensity intermittent running protocol. The results showed a higher increase in myoglobin levels and a more modest one in CK levels, with a return to the baseline values within 24 h for both parameters [66].

On a longer time span, CK and myoglobin levels were measured during a 12-day training period, with blood samples taken prior, at the 6-day mark, and on the 12th day at the end of the training period. CK values peaked on the 6th day, with a drop afterward, while myoglobin peaked on the 12th day [67].

By combining the results of both the cardiac biomarker measurements and of the CPET we can observe how biomarkers are also useful in the assessment of CRF, as they are released into the bloodstream when the cardiovascular stress is at higher values.

The main limitation of our study is represented by the small number of participants, which was not sufficient for more complex statistical analysis tests and could not offer sufficient data for further correlations (see Appendix A).

However, given the low number of publications and existing studies of this design, which combines the complete cardiovascular evaluation of athletes starting from the history, physical examination, 12-lead ECG and cardiac ultrasound and focusing on the combination of the CPET and cardiac biomarker measurements, it is definitely a starting point for further research and future studies. This is also supported by the significant statistical results which were obtained.

Our study offers a more complete approach than other studies, with the combination of CPET and biomarker measurements, and the established correlations so far encourage future research on larger groups, even though the biomarkers which suffered blood level changes (CK-MB, myoglobin, D-dimers) were not specific on their own for coronary diseases [37,38,39,40,41,48]. This association of CPET and biomarkers is also useful to be implemented in cardiac and respiratory rehabilitation evaluations, as shown by a 2021 study conducted by Wang et al., where the improvement of CHF patients’ parameters was monitored using these dynamics [68]. Apart from a higher number of subjects, serial measurements of cardiac biomarkers at more timepoints, especially at 12 and 24 h, would offer more indication of their full dynamics in relation to induced physical stress.

## 5. Conclusions

The current study showed that when subjected to an induced physical effort, biomarkers such as CK-MB, myoglobin, and D-dimers suffer changes in their blood levels. Additionally, between these biomarkers which suffered changes in relation to induced physical stress and the parameters which evaluated the functional capacity in the CPET, significant correlations were observed. This highlights the potential of the combined CPET and biomarker determinations for the functional evaluation of young professional athletes and the assessment of cardiovascular risk, alongside the 12-lead ECG and the cardiac ultrasound. However, additional research on a larger study group is needed to further ascertain these findings.

## Figures and Tables

**Table 1:** Electrocardiographic patterns in athletes and their interpretation (RBBB—right bundle branch block; AV—atrio-ventricular; PCV—premature ventricular contraction) (adapted from Sharma et al. [5]).

ECG Patterns in Athletes			References
*Normal*	*Borderline*	*Abnormal*	[5]
Increased QRS voltage Incomplete RBBB Early repolarization Black athlete repolarization variant Juvenile T wave pattern Sinus bradycardia Sinus arrhythmia Ectopic atrial rhythm Junctional escape rhythm 1st-degree atrioventricular block Mobitz Type I 2nd-degree atrioventricular block	Left axis deviation Left atrial enlargement Right axis deviation Right atrial enlargement Complete RBBB	T wave inversion ST-segment depression Pathologic Q waves Complete left bundle branch block Profound nonspecific intra-ventricular conduction delay Epsilon wave Ventricular pre-excitation Prolonged QT interval Brugada Type 1 pattern Profound sinus bradycardia Profound 1st-degree AV block Mobitz Type II 2nd-degree AV block 3rd-degree AV block Atrial tachyarrhythmias PVC Ventricular arrhythmias	

**Table 2:** Exercise-related major adverse cardiovascular events (MACE) [6].

Exercise-Related MACE	References
Sudden cardiac arrest (SCA) Sudden cardiac death (SCD) Acute coronary syndromes (ACS) Transient ischemic attacks (TIA) Cerebrovascular accidents (CVA) Supraventricular tachyarrhythmias	[6]

**Table 4:** Anthropometric and baseline BP and HR values.

Parameter	Mean	Standard Deviation
Age (years)	18.47	0.84
SBP baseline (mmHg)	120.47	6.97
DBP baseline (mmHg)	75.74	6.35
HR baseline (bpm)	63.68	13.55
Height (m)	1.77	0.06
Weight (kg)	72.89	7.26
BMI (kg/m^2^)	23.13	1.84

**Table 5:** TTE parameters.

Parameter	Mean	Standard Deviation
EDV (Teich, mL)	115.24	23.53
ESV (Teich, mL)	33.84	8.40
SV (Teich, mL)	81.50	19.42
EF (Teich, %)	70.42	5.99
FS (%)	40.19	4.85
SI (Teich, mL/m^2^)	42.90	9.96
IVSTd (mm)	10.17	1.49
LVIDd (mm)	49.22	4.33
LVPWTd (mm)	10.56	1.49
IVSTs (mm)	13.63	1.90
LVIDs (mm)	43.27	60.55
LVPWTs (mm)	16.54	2.27
LV MASSd (ASE, g)	234.63	50.18
LV MASSd Index (ASE, g/m^2^)	123.66	25.17
LV MASSs (ASE, g)	191.16	54.05
LV MASSs Index (ASE, g/m^2^)	103.95	25.46
E Vel (cm/s)	76.96	13.63
A Vel (cm/s)	44.15	8.08
E/A	1.78	0.38
A/E	0.58	0.13
DcT (s)	0.25	0.05
MVArea PHT (cm^2^)	4.51	6.45
PHT (s)	0.07	0.01
LVOT Diam (mm)	25.53	1.51
Ao Diam (mm)	30.44	2.30
LA Diam (mm)	27.88	3.82
LA/Ao	0.91	0.12

Abbreviations: EDV—end-diastolic volume; ESV—end-systolic volume; SV—stroke volume; EF—ejection fraction; FS—fractional shortening; SI—stroke-volume index; IVSTd—interventricular septum thickness at end-diastole; LVIDd—left ventricular internal dimension at end-diastole; LVPWTd—left ventricular posterior wall thickness at end-diastole; IVSTs—interventricular septum thickness at end-systole; LVIDs—left ventricular internal dimension at end-systole; LVPWTs—left ventricular posterior wall thickness at end-systole; LV MASSd—left ventricular mass at end-diastole; LV MASSd Index—left ventricular mass at end-diastole adjusted to body surface index; LV MASSs—left ventricular mass at end-systole; LV MASSs Index—left ventricular mass at end-systole adjusted to body surface index; E Vel—peak velocity of early diastolic mitral annular motion as determined by pulsed wave Doppler; A Vel—peak velocity of diastolic mitral annular motion as determined by pulsed wave Doppler; E/a—ratio of E to A; A/E—ratio of A to E; DcT—deceleration time MV area; PHT—mitral valve area at pressure half time; PHT—pressure half time; LVOT Diam—left ventricular outflow tract diameter; Ao Diam—aortic annulus diameter; LA Diam—left atrium diameter; LA/Ao—ratio of the left atrial dimension to the aortic annulus dimension.

**Table 6:** CPET parameters.

Parameter	Mean	Standard Deviation
VO_2_ max (mL/min)	3565.58	599.96
VO_2_ max body weight (mL/min/kg)	49.20	8.29
%VO_2_ max	106.89	17.13
VO_2_@AT (mL/min)	2558.84	703.16
VO_2_@AT body weight (mL/min/kg)	35.43	9.97
RER	1.12	0.08
VE/VCO_2_	23.54	3.37
ΔVO_2_/ΔWR (mL/min/Watt)	13.24	1.54
WR max (Watt)	249.68	25.36
%WR max	85.37	9.47
O_2__pulse (mL/beat)	21.28	3.55
%O_2__pulse	128.66	20.57
HR max (bpm)	170.89	11.28
%HR max	84.79	5.63
HR_rez (bpm)	107.21	13.75
SBP max (mmHg)	215.53	16.57
DBP max (mmHg)	83.42	4.42

Abbreviations: VO_2_ max—maximum oxygen uptake; VO_2_ max body weight—ratio of maximum oxygen uptake to body weight; %VO_2_ max—percentage of maximum oxygen uptake from the predicted value; VO_2_@AT—oxygen uptake at the anaerobic threshold; VO_2_@AT body weight—ratio of oxygen uptake at the anaerobic threshold to body weight; RER—respiratory exchange ratio; VE/VCO_2_—ventilatory equivalent for carbon dioxide; ΔVO_2_/ΔWR—slope of the relation VO_2_–power in W; WR max—maximum load; %WR max—percentage of maximum load from the predicted value; O_2__pulse—oxygen pulse; HR max—maximum heart rate; %HR max—percentage of maximum heart rate from the predicted value; HR_rez—heart rate reserve; SBP max—maximum systolic blood pressure; DBP max—maximum diastolic blood pressure.

**Table 7:** Biomarker variation analysis.

Parameter	Mean	Standard Deviation	Number of Subjects with Values above the Lower Detection Limit
Myoglobin baseline (ng/mL)	37.71	19.74	5
Myoglobin 3 h (ng/mL)	53.70	93.94	5
CK-MB baseline (ng/mL)	2.76	1.17	1
CK-MB 3 h (ng/mL)	2.86	1.40	3
D-dimer baseline (mg/L)	0.16	0.14	7
D-dimer 3 h (mg/L)	0.14	0.10	8

**Table 8:** Significant differences between the groups without changes (No) and with changes (Yes) in CK-MB values.

Parameter	CK-MB Change (No)	CK-MB Change (Yes)	*p*-Value
HR ECG (bpm)	54.50 (50.00–57.50)	67.00 (61.50–72.50)	0.047
VO_2_ max (mL/min)	3391.50 (2908.75–3907.50)	4148.00 (4116.00–4463.50)	0.008
Watt max (Watt)	241.50 (223.00–260.00)	285.00 (285.00–285.00)	0.014
Number of subjects	16	3	

**Table 9:** Significant differences between the groups without changes (No) and with changes (Yes) in D-dimers values.

Parameter	D-Dimers Change (No)	D-Dimers Change (Yes)	*p*-Value
%Watt max	87.50 (85.00–93.25)	84.00 (79.50–85.50)	0.035
Number of subjects	10	9	

**Table 10:** Significant differences between the groups without changes (No) and with changes (Yes) in myoglobin values.

Parameter	Myoglobin Change (No)	Myoglobin Change (Yes)	*p*-Value
E/A	1.90 (1.70–2.09)	1.57 (1.23–1.64)	0.036
A/E	0.53 (0.47–0.58)	0.640 (0.61–0.81)	0.036
VO_2_ @ AT (mL/min)	2688.00 (2211.25–3401.25)	2215.00 (1995.00–2431.00)	0.045
VO_2_ @ AT/body weight (mL/min/kg)	40.34 (31.91–46.16)	27.94 (23.47–35.15)	0.017
% WR max	87.00 (84.50–89.75)	82.00 (71.00-84.00)	0.045
% O_2_ Pulse	136.57 (123.38–150.21)	114.11 (96.70–137.10)	0.028
HR max (bpm)	165.00 (161.25–167.75)	183.00 (175.00–190.00)	0.013
% HR max	81.50 (80.00–83.75)	91.00 (87.00–94.00)	0.017
Number of subjects	12	7	

## Data Availability

Not applicable.

## Appendix A

This is a pilot study from a much larger research project in a doctoral study conducted within the “Grigore T. Popa” University of Medicine and Pharmacy in Iași, Romania, entitled “The evaluation of the correlations between the cardiac biomarkers and the risk of cardiovascular events during sustained physical effort”, under the direct coordination and supervision of Professor Habil. Florin Mitu, MD, PhD.

## References

[1] Makar O., Siabrenko G. (2018). Influence of Physical Activity on Cardiovascular System and Prevention of Cardiovascular Diseases (Review). Georgian Med. News.

[2] Jakovljevic D.G. (2018). Physical activity and cardiovascular aging: Physiological and molecular insights. Exp. Gerontol..

[3] Franklin B.A., Thompson P.D., Al-Zaiti S.S., Albert C.M., Hivert M.F., Levine B.D., Lobelo F., Madan K., Sharrief A.Z., Eijsvogels T.M.H. (2020). Exercise-related acute cardiovascular events and potential deleterious adaptations following long-term exercise training: Placing the risks into perspective-an update: A scientific statement from the American Heart Association. Circulation.

[4] Poddębska I., Kosielski P., Gałczyński S., Wranicz K., Cygankiewicz I., Kaczmarek K. (2020). ECG abnormalities in athletes as compare to healthy subjects. Pol. Merkur. Lek. Organ Pol. Tow. Lek..

[5] Sharma S., Drezner J.A., Baggish A., Papadakis M., Wilson M.G., Prutkin J.M., La Gerche A., Ackerman M.J., Borjesson M., Salerno J.C. (2018). International recommendations for electrocardiographic interpretation in athletes. Eur. Heart J..

[6] Pelliccia A., Sharma S., Gati S., Bäck M., Börjesson M., Caselli S., Collet J.P., Corrado D., Drezner J.A., Halle M. (2021). 2020 ESC Guidelines on sports cardiology and exercise in patients with cardiovascular disease. Eur. Heart J..

[7] Harmon K.G., Asif I.M., Maleszewski J.J., Owens D.S., Prutkin J.M., Salerno J.C., Zigman M.L., Ellenbogen R., Rao A.L., Ackerman M.J. (2015). Incidence, Cause, and Comparative Frequency of Sudden Cardiac Death in National Collegiate Athletic Association Athletes: A Decade in Review. Circulation.

[8] Drezner J.A., Harmon K.G., Marek J.C. (2014). Incidence of sudden cardiac arrest in Minnesota high school student athletes: The limitations of catastrophic insurance claims. J. Am. Coll. Cardiol..

[9] Maron B.J., Haas T.S., Ahluwalia A., Rutten-Ramos S.C. (2013). Incidence of cardiovascular sudden deaths in Minnesota high school athletes. Heart Rhythm.

[10] Harmon K.G., Asif I.M., Maleszewski J.J., Owens D.S., Prutkin J.M., Salerno J.C., Zigman M.L., Ellenbogen R., Rao A.L., Ackerman M.J. (2016). Incidence and Etiology of Sudden Cardiac Arrest and Death in High School Athletes in the United States. Mayo Clin. Proc..

[11] Toresdahl B.G., Rao A.L., Harmon K.G., Drezner J.A. (2014). Incidence of sudden cardiac arrest in high school student athletes on school campus. Heart Rhythm.

[12] Suárez-Mier M.P., Aguilera B., Mosquera R.M., Sánchez-de-León M.S. (2013). Pathology of sudden death during recreational sports in Spain. Forensic Sci. Int..

[13] Finocchiaro G., Papadakis M., Robertus J.L., Dhutia H., Steriotis A.K., Tome M., Mellor G., Merghani A., Malhotra A., Behr E. (2016). Etiology of Sudden Death in Sports: Insights from a United Kingdom Regional Registry. J. Am. Coll. Cardiol..

[14] Ullal A.J., Abdelfattah R.S., Ashley E.A., Froelicher V.F. (2016). Hypertrophic Cardiomyopathy as a Cause of Sudden Cardiac Death in the Young: A Meta-Analysis. Am. J. Med..

[15] Thiene G., Rizzo S., Schiavon M., Maron M.S., Zorzi A., Corrado D., Maron B.J., Basso C. (2019). Structurally Normal Hearts Are Uncommonly Associated with Sudden Deaths in Athletes and Young People. J. Am. Coll. Cardiol..

[16] Malhotra A., Sharma S. (2018). Outcomes of Cardiac Screening in Adolescent Soccer Players. N. Engl. J. Med..

[17] Hevia A.C., Fernández M.M., Palacio J.M., Martín E.H., Castro M.G., Reguero J.J. (2011). ECG as a part of the preparticipation screening programme: An old and still present international dilemma. Br. J. Sports Med..

[18] Fudge J., Harmon K.G., Owens D.S., Prutkin J.M., Salerno J.C., Asif I.M., Haruta A., Pelto H., Rao A.L., Toresdahl B.G. (2014). Cardiovascular screening in adolescents and young adults: A prospective study comparing the Pre-participation Physical Evaluation Monograph 4th Edition and ECG. Br. J. Sports Med..

[19] Drezner J.A., Prutkin J.M., Harmon K.G., O’Kane J.W., Pelto H.F., Rao A.L., Hassebrock J.D., Petek B.J., Teteak C., Timonen M. (2015). Cardiovascular screening in college athletes. J. Am. Coll. Cardiol..

[20] Drezner J.A., Owens D.S., Prutkin J.M., Salerno J.C., Harmon K.G., Prosise S., Clark A., Asif I.M. (2016). Electrocardiographic Screening in National Collegiate Athletic Association Athletes. Am. J. Cardiol..

[21] Price D.E., McWilliams A., Asif I.M., Martin A., Elliott S.D., Dulin M., Drezner J.A. (2014). Electrocardiography-inclusive screening strategies for detection of cardiovascular abnormalities in high school athletes. Heart Rhythm.

[22] Rizzo M., Spataro A., Cecchetelli C., Quaranta F., Livrieri S., Sperandii F., Cifra B., Borrione P., Pigozzi F. (2012). Structural cardiac disease diagnosed by echocardiography in asymptomatic young male soccer players: Implications for pre-participation screening. Br. J. Sports Med..

[23] Tomkinson G.R., Lang J.J., Blanchard J., Léger L.A., Tremblay M.S. (2019). The 20-m Shuttle Run: Assessment and Interpretation of Data in Relation to Youth Aerobic Fitness and Health. Pediatr. Exerc. Sci..

[24] Guazzi M., Bandera F., Ozemek C., Systrom D., Arena R. (2017). Cardiopulmonary Exercise Testing: What Is its Value. J. Am. Coll. Cardiol..

[25] Tran D. (2018). Cardiopulmonary Exercise Testing. Methods Mol. Biol..

[26] Arena R., Canada J.M., Popovic D., Trankle C.R., Del Buono M.G., Lucas A., Abbate A. (2020). Cardiopulmonary exercise testing—Refining the clinical perspective by combining assessments. Expert Rev. Cardiovasc. Ther..

[27] Gibson M.E., Gray K. (2019). Exercise Testing. Curr. Sports Med. Rep..

[28] Mezzani A. (2017). Cardiopulmonary Exercise Testing: Basics of Methodology and Measurements. Ann. Am. Thorac. Soc..

[29] Foster C. (2017). Is There Risk in Exercise Testing of Athletes. Int. J. Sports Physiol. Perform..

[30] Guazzi M., Adams V., Conraads V., Halle M., Mezzani A., Vanhees L., Arena R., Fletcher G.F., Forman D.E., Kitzman D.W. (2012). EACPR/AHA Scientific Statement. Clinical recommendations for cardiopulmonary exercise testing data assessment in specific patient populations. Circulation.

[31] Löllgen H., Leyk D. (2018). Exercise Testing in Sports Medicine. Dtsch. Arztebl. Int..

[32] Katrukha I.A. (2013). Human cardiac troponin complex. Structure and functions. Biochemistry.

[33] Van der Velden J., Stienen G.J.M. (2019). Cardiac Disorders and Pathophysiology of Sarcomeric Proteins. Physiol. Rev..

[34] Bódi B., Oláh A., Mártha L., Tóth A., Radovits T., Merkely B., Papp Z. (2021). Exercise-induced alterations of myocardial sarcomere dynamics are associated with hypophosphorylation of cardiac troponin I. Rev. Cardiovasc. Med..

[35] Kosowski M., Młynarska K., Chmura J., Kustrzycka-Kratochwil D., Sukiennik-Kujawa M., Todd J.A., Jankowska E.A., Banasiak W., Reczuch K., Ponikowski P. (2019). Cardiovascular stress biomarker assessment of middle-aged non-athlete marathon runners. Eur. J. Prev. Cardiol..

[36] Samaha E., Avila A., Helwani M.A., Ben Abdallah A., Jaffe A.S., Scott M.G., Nagele P. (2019). High-Sensitivity Cardiac Troponin After Cardiac Stress Test: A Systematic Review and Meta-Analysis. J. Am. Heart Assoc..

[37] Fan J., Ma J., Xia N., Sun L., Li B., Liu H. (2017). Clinical Value of Combined Detection of CK-MB, MYO, cTnI and Plasma NT-proBNP in Diagnosis of Acute Myocardial Infarction. Clin. Lab..

[38] Tota Ł., Piotrowska A., Pałka T., Morawska M., Mikuľáková W., Mucha D., Żmuda-Pałka M., Pilch W. (2019). Muscle and intestinal damage in triathletes. PLoS ONE.

[39] Bjørnsen T., Wernbom M., Paulsen G., Berntsen S., Brankovic R., Stålesen H., Sundnes J., Raastad T. (2021). Frequent blood flow restricted training not to failure and to failure induces similar gains in myonuclei and muscle mass. Scand. J. Med. Sci. Sports.

[40] Safdar B., Bezek S.K., Sinusas A.J., Russell R.R., Klein M.R., Dziura J.D., D’Onofrio G. (2014). Elevated CK-MB with a normal troponin does not predict 30-day adverse cardiac events in emergency department chest pain observation unit patients. Crit. Pathw. Cardiol..

[41] Sahadeo P.A., Dym A.A., Berry L.B., Bahar P., Singla A., Cheta M., Bhansali R., LaVine S., Laser J., Richman M. (2021). The Best of Both Worlds: Eliminating Creatine Kinase-Muscle/Brain (CK-MB) Testing in the Emergency Department Leads to Lower Costs Without Missed Clinical Diagnoses. Cureus.

[42] Zhang Z.L., Li R., Yang F.Y., Xi L. (2018). Natriuretic peptide family as diagnostic/prognostic biomarker and treatment modality in management of adult and geriatric patients with heart failure: Remaining issues and challenges. J. Geriatr. Cardiol..

[43] Vassalle C., Masotti S., Lubrano V., Basta G., Prontera C., Di Cecco P., Del Turco S., Sabatino L., Pingitore A. (2018). Traditional and new candidate cardiac biomarkers assessed before, early, and late after half marathon in trained subjects. Eur. J. Appl. Physiol..

[44] Roca E., Nescolarde L., Lupon J., Barallat J., Januzzi J.L., Liu P., Cruz Pastor M., BayesGenis A. (2017). The dynamics of cardiovascular biomarkers in non-elite marathon runners. J. Cardiovasc. Transl. Res..

[45] Cocking S., Landman T., Benson M., Lord R., Jones H., Gaze D., Thijssen D.H.J., George K. (2017). The impact of remote ischemic preconditioning on cardiac biomarker and functional response to endurance exercise. Scand. J. Med. Sci. Sports.

[46] Pearson M.J., King N., Smart N.A. (2018). Effect of exercise therapy on established and emerging circulating biomarkers in patients with heart failure: A systematic review and meta-analysis. Open Heart.

[47] Limkakeng A.T., Leahy J.C., Griffin S.M., Lokhnygina Y., Jaffa E., Christenson R.H., Newby L.K. (2018). Provocative biomarker stress test: Stress-delta N-terminal pro-B type natriuretic peptide. Open Heart.

[48] Koch V., Biener M., Müller-Hennessen M., Vafaie M., Staudacher I., Katus H.A., Giannitsis E. (2020). Diagnostic performance of D-dimer in predicting venous thromboembolism and acute aortic dissection. Eur. Heart J. Acute Cardiovasc. Care.

[49] Almorad A., Ohanyan A., Pintea Bentea G., Wielandts J.Y., El Haddad M., Lycke M., O’Neill L., Morissens M., De Keyzer E., Nguyen T. (2021). D-dimer blood concentrations to exclude left atrial thrombus in patients with atrial fibrillation. Heart.

[50] Miller M.J., Maier C.L., Duncan A., Guarner J. (2021). Assessment of Coagulation and Hemostasis Biomarkers in a Subset of Patients with Chronic Cardiovascular Disease. Clin. Appl. Thromb. Hemost..

[51] Lombardo B., Izzo V., Terracciano D., Ranieri A., Mazzaccara C., Fimiani F., Cesaro A., Gentile L., Leggiero E., Pero R. (2019). Laboratory medicine: Health evaluation in elite athletes. Clin. Chem. Lab. Med..

[52] Pepera G., Hadjiandrea S., Iliadis I., Sandercock G.R.H., Batalik L. (2022). Associations between cardiorespiratory fitness, fatness, hemodynamic characteristics, and sedentary behaviour in primary school-aged children. BMC Sports Sci. Med. Rehabil..

[53] Olekšák F., Dvoran P., Jakušová Ľ., Ďurdík P., Igaz M., Bánovčin P. (2021). Reference Values for Cardiopulmonary Exercise Testing in Young Male Slovak Athletes. Acta Med..

[54] Mahanty A., Xi L. (2020). Utility of cardiac biomarkers in sports medicine: Focusing on troponin, natriuretic peptides, and hypoxanthine. Sports Med. Health Sci..

[55] Sedaghat-Hamedani F., Kayvanpour E., Frankenstein L., Mereles D., Amr A., Buss S., Keller A., Giannitsis E., Jensen K., Katus H.A. (2015). Biomarker changes after strenuous exercise can mimic pulmonary embolism and cardiac injury—A metaanalysis of 45 studies. Clin. Chem..

[56] Hosseini S.M., Azizi M., Samadi A., Talebi N., Gatterer H., Burtscher M. (2018). Impact of a Soccer Game on Cardiac Biomarkers in Adolescent Players. Pediatr. Exerc. Sci.

[57] Bjørkavoll-Bergseth M., Erevik C.B., Kleiven Ø., Eijsvogels T.M.H., Skadberg Ø., Frøysa V., Wiktorski T., Auestad B., Edvardsen T., Moberg Aakre K. (2021). Determinants of Interindividual Variation in Exercise-Induced Cardiac Troponin I Levels. J. Am. Heart Assoc..

[58] Scherr J., Braun S., Schuster T., Hartmann C., Moehlenkamp S., Wolfarth B., Pressler A., Halle M. (2011). 72-h kinetics of high-sensitive troponin T and inflammatory markers after marathon. Med. Sci. Sports Exerc..

[59] Marshall L., Lee K.K., Stewart S.D., Wild A., Fujisawa T., Ferry A.V., Stables C.L., Lithgow H., Chapman A.R., Anand A. (2020). Effect of Exercise Intensity and Duration on Cardiac Troponin Release. Circulation.

[60] Costache A.-D., Costache I.-I., Miftode R.-Ș., Stafie C.-S., Leon-Constantin M.-M., Roca M., Drugescu A., Popa D.-M., Mitu O., Mitu I. (2021). Beyond the Finish Line: The Impact and Dynamics of Biomarkers in Physical Exercise—A Narrative Review. J. Clin. Med..

[61] Miftode R.-S., Constantinescu D., Cianga C.M., Petris A.O., Timpau A.-S., Crisan A., Costache I.-I., Mitu O., Anton-Paduraru D.-T., Miftode I.-L. (2021). A Novel Paradigm Based on ST2 and Its Contribution towards a Multimarker Approach in the Diagnosis and Prognosis of Heart Failure: A Prospective Study during the Pandemic Storm. Life.

[62] Hammadah M., Kim J.H., Tahhan A.S., Kindya B., Liu C., Ko Y.A., Al Mheid I., Wilmot K., Ramadan R., Alkhoder A. (2018). Use of High-Sensitivity Cardiac Troponin for the Exclusion of Inducible Myocardial Ischemia: A Cohort Study. Ann. Intern. Med..

[63] Walter J.E., Honegger U., Puelacher C., Mueller D., Wagener M., Schaerli N., Strebel I., Twerenbold R., Boeddinghaus J., Nestelberger T. (2018). Prospective Validation of a Biomarker-Based Rule Out Strategy for Functionally Relevant Coronary Artery Disease. Clin. Chem..

[64] Lima B.B., Hammadah M., Kim J.H., Uphoff I., Shah A., Levantsevych O., Almuwaqqat Z., Moazzami K., Sullivan S., Ward L. (2020). Relation of High-sensitivity Cardiac Troponin I Elevation with Exercise to Major Adverse Cardiovascular Events in Patients with Coronary Artery Disease. Am. J. Cardiol..

[65] Chamera T., Spieszny M., Klocek T., Kostrzewa-Nowak D., Nowak R., Lachowicz M., Buryta R., Ficek K., Eider J., Moska W. (2015). Post-Effort Changes in Activity of Traditional Diagnostic Enzymatic Markers in Football Players’ Blood. J. Med. Biochem..

[66] Joo C.H. (2015). Development of a non-damaging high-intensity intermittent running protocol. J. Exerc. Rehabil..

[67] Radzimiński Ł., Jastrzębski Z., López-Sánchez G.F., Szwarc A., Duda H., Stuła A., Paszulewicz J., Dragos P. (2020). Relationships between Training Loads and Selected Blood Parameters in Professional Soccer Players during a 12-Day Sports Camp. Int. J. Environ. Res. Public Health.

[68] Wang Y., Cao J., Kong X., Wang S., Meng L., Wang Y. (2021). The effects of CPET-guided cardiac rehabilitation on the cardiopulmonary function, the exercise endurance, and the NT-proBNP and hscTnT levels in CHF patients. Am. J. Transl. Res..

